# What should be considered if you decide to build your own mathematical model for predicting the development of bacterial resistance? Recommendations based on a systematic review of the literature

**DOI:** 10.3389/fmicb.2015.00352

**Published:** 2015-04-29

**Authors:** Maria Arepeva, Alexey Kolbin, Alexey Kurylev, Julia Balykina, Sergey Sidorenko

**Affiliations:** ^1^Faculty of Applied Mathematics and Control Processes, St. Petersburg State UniversitySt. Petersburg, Russia; ^2^Faculty of Medicine, First Pavlov State Medical University of St. PetersburgSt. Petersburg, Russia; ^3^Department of Molecular Microbiology and Epidemiology, Scientific Research Institute of Childhood InfectionsSt. Petersburg, Russia; ^4^Department of Medical Microbiology, North-Western State Medical University named after I.I. MechnikovSt. Petersburg, Russia

**Keywords:** bacterial resistance, mathematical model, antibiotics

## Abstract

Acquired bacterial resistance is one of the causes of mortality and morbidity from infectious diseases. Mathematical modeling allows us to predict the spread of resistance and to some extent to control its dynamics. The purpose of this review was to examine existing mathematical models in order to understand the pros and cons of currently used approaches and to build our own model. During the analysis, seven articles on mathematical approaches to studying resistance that satisfied the inclusion/exclusion criteria were selected. All models were classified according to the approach used to study resistance in the presence of an antibiotic and were analyzed in terms of our research. Some models require modifications due to the specifics of the research. The plan for further work on model building is as follows: modify some models, according to our research, check all obtained models against our data, and select the optimal model or models with the best quality of prediction. After that we would be able to build a model for the development of resistance using the obtained results.

## Introduction

In spite of significant advances in the development of antibiotics (AB) and antibacterial drugs in general, infectious diseases remain a major cause of morbidity and mortality worldwide (Whitby et al., 2001; Schwaber and Carmeli, 2007; Macgowan, 2008; Spellberg et al., 2008). Antibiotic resistance (ABR) is considered to be a key factor in these unsatisfactory outcomes. Its increase has been recognized as a real threat to the global population's health, which was reflected in the “Strategies for global surveillance of antimicrobial resistance” adopted by the World Health Organization (World Health Organization, 2001). It is impossible to prevent the development and spread of all ABR due to bacteria's innate property of adapting to a changing environment, however, the rate of increase of ABR needs to be reduced to prevent a greater problem. Meanwhile, it seems viable to slow down the spread of ABR and maintain the effectiveness of existing etiotropic treatment (World Health Organization, 2001; Mölstad et al., 2008). Good, appropriate approaches of antimicrobial stewardship are thought to be pivotal in improving the resistance situation. The aim of this review was to evaluate mathematical models which link antibiotic usage to resistance, for this purpose, a systematic search and analytic review of the literature was performed.

## Materials and methods

The method of this study involved a systematic search and subsequent reviewing of publications relating to different mathematical models of ABR. The search for publications that came out during the last 10 years (from 03.11.2003 to 03.11.2013) was performed in the MEDLINE, Web of Science and Scopus databases.

### Selection of studies and search criteria

The search for relevant publications was performed in the PubMed database using the following search request: determinist^*^ OR stochast^*^ OR discrete OR mathematic^*^ OR simulat^*^ AND model^*^ AND resist^*^ AND (“2003/11/03”[PDat]: “2013/10/30”[PDat] AND “humans” [MeSHTerms]). The following inclusion and exclusion criteria were developed and applied. *Inclusion criteria*: any model describing ABR. *Exclusion criteria*: non-bacterial resistance (caused by viruses, fungi, mycobacteria, protozoa, etc.); studies of ABP based on animal models only; models of resistance transmission *in vitro* between strains; pharmacokinetic/pharmacodynamics models; models of resistant strains spreading in a human population under conditions of a lack of AB.

### Selection and analysis of publications

**Figure 1:**
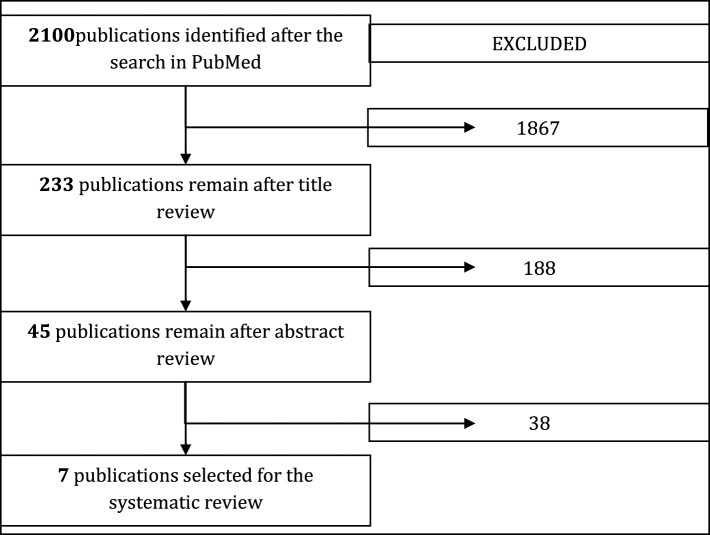
**Diagram of publication selection for systematic review**. This paper reviews the seven models which remained after the filtering process.

The primary selection of publications was assessed by five independent reviewers: two mathematicians, two clinical pharmacologists, and one microbiologist. Each publication selected by the search query was assessed by the reviewers in terms of the inclusion and exclusion criteria using the following scheme: assessment after reading a title; assessment after reading an abstract; assessment after reading the full text. Final agreement on the publication list for further analysis and review was made on a consensus basis. In case of disagreements voting combined with formal Delphi methodologies (reaching a consensus between the experts by two rounds with 85% agreement) was used. The selection diagram is presented in Figure 1.

### Preliminary conclusions

In most studies selected at the stage of abstract review the development and spread of ABR was considered using the epidemiological approach as a simplified means of describing the transmission of communicable disease through individuals without considering the impact of antibiotics. We reviewed only models where AB consumption was introduced as an independent parameter, because we consider the process of developing resistance as a function of time and antibiotic consumption. That is why some papers were excluded at the stage of full text analysis (Bergstrom et al., 2004; Miller et al., 2004; Boni and Feldman, 2005; Huovinen, 2005; Mera, 2005; Reluga, 2005; Webb et al., 2005; Levin and Rozen, 2006; Schinazi, 2006; D'Agata et al., 2007; Zalounina et al., 2007; Drovandi and Pettitt, 2008; Pienaar et al., 2009; Artalejo et al., 2010; Haber et al., 2010; Kardas-Sloma et al., 2011; Kouyos et al., 2011; Opatowski et al., 2011; Wang et al., 2011; Banks et al., 2012; Chamchod and Ruan, 2012a,b; Deng et al., 2012; Hurford et al., 2012; Joyner et al., 2012; Sotto and Lavigne, 2012; Yahdi et al., 2012; Horsburgh et al., 2013; Lee et al., 2013a,b; Worby et al., 2013). The seven remaining models were classified according to the type of link between resistance and AB consumption. Three major classes of model and one additional class were created (Table 1).

**Table 1:** **Classes of mathematical model**.

**Class**	**Method of introducing AB into the model**	**References**	**Description**
1.	DDD^*^	Berger et al., 2004; Aldrin et al., 2013	Time series, regression, the biological process is not considered in detail
2.	Proportions of patients receiving treatment	D'Agata et al., 2005	Differential equations, the biological process is not considered in detail
3.	Dose (as a rate of growth suppression/the drug kill rate) and duration of therapy	D'Agata et al., 2008; Friedman et al., 2010	Differential equations, the biological process is considered in detail
4.	Difficult to classify because of the specific approach	Geli et al., 2006; Sun et al., 2010	Seasonal correlation between antibiotic consumption and resistance. Artificial neural network.

* *DDD, Defined daily dose*.

We examined each model in detail and assessed the suitability and successfulness of each class in addressing the research question.

## Results

### Class 1. DDD as an independent parameter to enter the antibiotic into the model

#### Model 1. Antibiotic resistance in hospitals: a ward-specific random effect model in a low antibiotic consumption environment (Aldrin et al., 2013)

##### Description of the model 1

This study quantified the impact of antibiotic consumption on the changes of *Pseudomonas aeruginosa* resistance in three Norwegian hospitals between January 2001 and December 2006. The main antibiotics under consideration were carbapenems (meropenem and imipenem), tandpenicillins (ampicillin/pivampicillin and mecillinam/pivmecillinam) to which *P. aeruginosa* is resistant. In particular, the study considered the relationship between changes in *P. aeruginosa* resistance and changes in antibiotic consumption. The main questions were:

1. How is the IR of *P. aeruginosa* affected by the use of antibacterial agents to which the microbe is resistant?
2. How is the proportion of *P. aeruginosa* non-susceptible to meropenem influenced by the use of carbapenems?

Antibiotic consumption was expressed as defined daily doses per 100 bed-days and microbiology data were obtained from the microbiological laboratories' databases. Thus, the following data were collected for each ward and month:

1. the incidence of *P. aeruginosa;*
2. the incidence of *P. aeruginosa* where susceptibility testing against meropenem was performed;
3. the incidence of *P. aeruginosa* resistant to meropenem;
4. the number of bed-days.

Subsequently, the following values were calculated: (1) the IR of *P. aeruginosa*, that is, the number of patients from whom the bacterium was isolated per 100 bed-days; (2) the proportion of *P. aeruginosa* resistant to meropenem, that is, the number of patients from whom the resistant variant was isolated divided by the total number of patients tested (binomial distribution).

The main approach of modeling the spread of resistance used in the model is the regression and autoregression analysis (autoregressive part with different lags was implemented for the resistance, regressive part—for the consumptions of AB with different lags and different confounders, like epidemic or holiday variables). Also model was tested for seasonality. Selection of the optimal model was carried out by Schwartz criterion. The simulation of the resistance at various levels of consumptions was made after finding the optimal model.

#### Model analysis

##### Preliminary conclusions

The class of regression models described in this paper commonly used in the study of time series. Authors specify resistance with logarithmic link (the model for incidence rate of resistant microbes) and logit link (the model for proportion of resistant bacteria). Autoregressive and regressive parts are introduced with different lags, so the authors get a lot of sub-models, because different lags for autoregressive part are combined with different lags for AB. The decision on whether to consider seasonality and confounders also increases the number of sub-models. To reduce the number of coefficients for different lags of AB consumption, authors describe these coefficients by the polynomial of lags of order 2. It would be correct to consider different set of AB in model, but it increased greatly the number of sub-models, so authors considered all AB simultaneously. Months where no susceptibility tests were performed were treated separately by the introduction of the new coefficient. Finally, all sub-models were tested using Bayesian information criterion. Unfortunately, this method of validation does not exclude the overfitting of the model, so we consider it appropriate to introduce the second step for validation. That is to break up all data sets into three subsets—raining set for finding coefficients, product set to exclude overfitted sub-models, and a test set to estimate the accuracy of forecast.

#### Model 2. Generalized additive model (GAM) demonstrates fluoroquinolone use/resistance relationships for *Staphylococcus aureus* (Berger et al., 2004)

##### Description of model 2

GAM was applied in this research to study fluoroquinolone use and the incidence of fluoroquinolone-resistance in *S. aureus*. Materials for microbiological studies were provided by data from four hospitals in Marseille taken over a period of 3 years (1997–2000). The information system in all four hospital pharmacies provided data on antimicrobials (amount in grams by class and formulation) prescribed starting from January 1, 1997, 6 months prior to the start of the microbiology study period. Data was obtained on fluoroquinolones (ciprofloxacine, ofloxacine, and pefloxacine), penicillins with β-lactamase inhibitors (amoxicillin/clavulanic acid, ticarcillin/clavulanic acid, and piperacillin/tazobactam), third-generation cephalosporins (cefotaxime, ceftriaxone, and ceftazidime), gentamicin, oxacillin, and rifampicin. The total amounts of AB consumed in grams each day were converted into numbers of defined daily doses (DDD), using the average maintenance dose of each drug as recommended by the manufacturers. The possibility of delayed effects from AB consumed was also considered. Monthly numbers of patient-days were obtained from each hospital's admission department to provide a measure of the risk of patients becoming infected or colonized. To quantify the association between incidence of resistance and antibiotic use, the authors applied the GAM, adapted to time series. A temporal analysis of the monthly variations of DDD number and the incidence was performed.

#### Model analysis

##### Preliminary conclusions

The model was based on a similar method as in model 1, but authors also considered the non-parametric smoothing for antibiotic use, that was performed by locally weighted scatterplot smoothing (LOESS). Autoregressive and regressive parts were introduced by the same way as in model 1. As to confounders, authors used a “holiday” variable (to control the lower occupation rates of beds in hospitals over public holidays) and “epidemic” binary variable (to control its potential confusing effect). A trend and seasonality were controlled by a spline function of a “month” variable. Binary variables were created to control possible variations between winter and summer. The criterion used to find the optimum model is Akaike's information criterion (AIC) The effect of therapy was not considered. In the first article the authors went further and examined the effect of changes in AB consumption (proportionally increasing or decreasing) and presented the quantitative results, something which was lacking in this study. Different sets of AB were considered in this model (unlike model 1), that increase the number of sub-models. To solve this problem the AB was introduced successively to each other with simultaneous estimation by AIC. Since the model 1 and model 2 and similar, the comments about validation are also similar. We would like to add one more comment about trend and autoregressive part, that is the opportunity to double count a trend into the autoregressive part. Therefore, it is necessary to consider options with and without the trend.

### Class 2. The fraction of patients receiving AB treatment serves as an independent parameter to include antibiotic use in the model

#### Model 3. A mathematical model quantifying the impact of antibiotic exposure and other interventions on the endemic prevalence of vancomycin-resistant *Enterococci* (D'Agata et al., 2005)

##### Description of the model

A model was developed to quantify the contribution of antibiotic exposure and other factors to the spread of vancomycin-resistant *Enterococci* (VRE) in the hospital setting. Patients were divided into four groups: patients colonized with (VRE) receiving antibiotic treatment; patients colonized with (VRE) who were not receiving antibiotic treatment; uncolonized patients receiving antibiotics and uncolonized patients who were not receiving antibiotics. Baseline parameter estimates were derived from pharmacological databases, infection control databases and clinical databases. It was assumed that patients colonized with VRE and receiving antibiotics were more likely to transmit VRE to other patients. Simulations were performed to quantify the impact of starting or discontinuing antibiotic treatment in different groups of patients either colonized or uncolonized with VRE on the incidence of VRE over time. The necessary data were obtained by analyzing computerized infection control records, which monitor the number of patients admitted to the hospital with a history of VRE, as well as pharmacy records (microbiological data). Values for average duration of hospital stay were obtained from the hospital administrative data. Simulation modeling was undertaken so as to estimate the impact of the following independent variables on the development of resistance:

1. the number of colonized and uncolonized patients who received AB;
2. the number of colonized and uncolonized patients who stopped receiving AB;
3. length of hospital stay of colonized patients;
4. hand hygiene among health care workers;
5. the ratio of health care workers to patients.

Each group is defined by a differential equation, describing the changes in different groups of patients over time (colonized/uncolonized, receiving AB/not receiving).

#### Model analysis

##### Preliminary conclusions

In this model, both patients colonized or uncolonized with VRE were included. The influence of antibiotics (fraction of patients) on resistance in the model was not described analytically and was introduced during the simulation analysis. As a result of generating simulations which considered different fractions of patients receiving antibiotics, the authors were able to track the main trends in the changes of resistance (fractions of patients colonized with resistant strains).

### Class 3. Dependency between resistance, antibiotic dosage (as a rate of growth suppression) and length of treatment

#### Model 4. A model of drug resistance with infection by health care workers (Friedman et al., 2010)

##### Description of the model

The purpose of this study is mathematical modeling of strain evolution among patients in hospitals. The model assumes that during 6 weeks of the simulation time of the model patients do not enter or leave the hospital. Also, the following assumptions were made:

1. the immune system of the patients acts to reduce the bacterial population;
2. non-resistant strains mutate into resistant ones at a certain rate;
3. infection is transmitted between personnel and patients at a certain rate.

The model is based on a series of differential equations and is not validated in a clinical setting.

#### Model analysis

##### Preliminary conclusions

The model is based on a differential equation of the dependence of the non-resistant and resistant strains on AB dosage. The following variables were included as factors in the model:

1. the rate of bacterial growth;
2. the immune response;
3. the drug kill rate (i.e., drug response, the reduction ratio of the number of bacteria) the transmission between patients and HCWs (the probability of transfer of bacteria).

AB dosage was categorized as either low, medium, or high and the graphs were made for different durations of therapy and different levels of consumption (low, medium, high). Thus, there was a rough estimate of the impact of AB consumption, only in terms of categories; there was no rigorous analytical link between number of bacteria and amount of consumption. The model was not validated in a real-world clinical setting and it did not use any experimental data for assessment of the parameters. Its main conclusion was that the longer treatment lasts and the higher AB dosage is, the lower the level of resistance is. Perhaps, the optimal treatment that the authors found is only a local minimum and resistance will grow again in the long-term, because 6 weeks is a very short period for simulation. The mechanism of mutations in this study was discussed only in context of AB, i.e., it was supposed that mutation occurs only if a bacterium is affected by AB. As mentioned earlier, the reporting period of time was very short, which excludes the possibility to assess the impact of delay consumption. The conclusions based on the results of modeling are of interest. If the main conclusions do not change significantly after revision of the model and substitution of our data, this approach deserves our attention.

#### Model 5. The impact of different antibiotic regimens on the emergence of antimicrobial-resistant bacteria (D'Agata et al., 2008)

##### Description of the model

This model contained three main parameters which are hypothesized to regulate the development of resistance: immune response, horizontal gene transfer, and treatment regimen. The model of bacteria within a host was considered in order to find optimal treatment which could restrain ABR. Differential equations were used. The authors take into account the effect of treatment with different durations of therapy, earlier or later start of therapy, and simultaneous consumption of several AB. The research included the following sub-models. **Model with immune response and single AB treatment [sub-model (1)]**. An ideal setting was modeled where a bacterial population within a host was treated with the appropriate AB. Since bacterial growth is impacted by availability of nutrients, a logistic curve was used to describe the dynamics of the population. It was assumed that the bacteria were dying under the influence of AB at steady rate which was proportional to their population density. Immunity is interpreted as the ability to react to bacteria, suppress their growth and destroy them by means of leucocytes. It was suggested that the killing rate of bacteria by leucocytes could be described by the Monod function. In this sub-model the authors considered only the minimal infecting dose, i.e., the threshold number of bacteria required to overcome immunity.

#### Model of the development of resistance to a single AB through horizontal gene transfer [sub-model (2)]

This model assumes that the AMR of bacteria can be intrinsic or acquired through mutation and acquisition of new AMR genes. The latter mechanism occurs through horizontal transfer of such genetic elements as plasmids, integrons, and transposons.

#### Model with different AB treatment and multidrug-resistant strains [sub-model (3)]

Sub-model 3 was based on sub-model 2 and extended through the assumption of multidrug-resistance of bacteria and modeling for different treatment regimens.

#### Model analysis

##### Preliminary conclusions

The authors created a dynamic model of the development of infection (as the bacterial load) within one host depending on the immune response, horizontal gene transfer, and presence of a strain (either resistant or sensitive to an antibiotic). The influence of AB was represented in the model through the bacterial mortality rate in the presence or absence of AB. More precisely, AB usage itself as a parameter was not included in the model; one can assess its effect only by simulations with different mortality rates. On the other hand, the mortality rate can be considered as being coefficiently linked with drug dosage, which produces the given mortality in bacteria population. Due to this assumption, and the fact that this study assessed the effect of treatment duration this study was classified as one where the effect of AB is introduced through its dosage and the duration of therapy.

### Additional models without class reference

#### Model 6. Seasonality and temporal correlation between community antibiotic use and resistance in the United States (Sun et al., 2010)

##### Description of the model 6

A study of the seasonal dependence between antibiotic prescription and resistance was conducted in the United States from 1999 to 2007. The study used the data on retail antibiotic sales obtained through IMS Health's Xponent database (Campos et al., 2007). The data from this source cover more than 70% of all antibiotics prescribed in the United States. The mass of data was broken down by prescription month into 5 major groups:

1. aminopenicillins
2. quinolones
3. macrolides/lincosamides
4. trimerthoprim/sulfamethoxazoles
5. tetracyclines.

Data on resistance were obtained from The Surveillance Network Database-USA (Sun et al., 2010), an electronic database with the susceptibility tests results for *Escherichia coli* and *methicillin-resistant Staphylococcus aureus* (MRSA) from more than 300 laboratories in the USA. Trends of the time series which mapped resistance and use of antibiotics were evaluated with LOESS method (locally weighted scatter plot smoothing). To convert the data into the form of time series with independent identically distributed random variables, the Box-Jenkins approach to time-series modeling was used.

#### Model analysis

##### Preliminary conclusions

To assess antibiotic consumption the amount of prescriptions was used instead of average daily dosage. Because of this, it is difficult to compare the results of this model with other studies. Moreover, this approach does not consider the dosage and duration of antibiotics therapy as independent parameters.

This model contains autocorrelation elements and is based on an ARIMA framework. However, this does not allow the model to define the cause and effect relationship between antibiotic resistance and drug usage. Moreover, this approach does not give any numerical results. Still, these results may be useful in finding the lag (as an alternative method for models of Class 1).

#### Model 7. Modeling pneumococcal resistance to penicillin in southern Sweden using artificial neural networks (Geli et al., 2006)

##### Description of the model

In this study a model based on an Artificial Neural Network (ANN) approach was developed to describe and predict the spread of penicillin-resistant pneumococci (PRP) as a function of antibiotic consumption and a number of confounders. Data on monthly incidence of PRP in 32 municipalities in the south of Sweden from 1997 to 2003 were analyzed. Use of neural networks suggests that there are inputs for which the result is known (these data are used for training the network as well as for checking the correctness of its performance). Typically, two-thirds of all known data is taken as the training set (on these data, the system is configured), and one-third is taken as the testing set (on these data the system is checked for errors). In this paper input information was taken from historical data for 1997–2003. The data from 1997 to 2000 on antibiotic consumption in the south of Sweden were taken as the training set, and information from 2001 to 2003—as a testing set to verify proper performance of the model.

Antibiotic consumption data were obtained from the database of the National Corporation of Swedish Pharmacies (Apoteket AB).

#### Model analysis

##### Preliminary conclusions

ANNs represent a wide class of computational methods for building flexible models, including linear regression, data reduction models, and nonlinear dynamical systems. Models in this paper make good predictions when checked against the actual data from 2001 to 2003. The main result of this study is that antibiotic usage and the incidences of PRP in the past do not have a significant impact on the incidence of PRP. One of the problems with the ANN topology used in this model is the sigmoid function (definition interval from 0 to 1) in the last layer. This means that the resulting predictions will vary in the range from the minimum to the maximum number of PRP cases (in the training data set), which is not very realistic. This problem may be solved by using a linear or inverse sigmoid function instead of the sigmoid function. On the other hand, using a bounded transfer function can lead to more conservative predictions. It would be interesting to conduct a comparative analysis of simulation results for the models of antibiotic resistance emergence obtained at different transfer functions.

## Discussion

Our objective was to identify and review models which looked at the factors which affect and determine antibiotic resistance. After a literature search to identify published models of this sort, we undertook a critical review of different approaches and model structures. The end purpose of this exercise is to increase our knowledge in building a functioning model that makes it possible to predict the development of resistance and control its dynamics. To achieve this goal, first of all, we need to understand what parameters should be included in the model. Also it seems most appropriate to use not one but several models which describe resistance, because the average forecast of several models can be more accurate than forecast of only one. We investigated models described above and now we offer our opinion as to which of these models under which conditions should be used to build your own predictive mathematical model of acquired resistance.

Initially, after analyzing the two models in class 1 that are similar in terms of mathematical approach [model 1 (Aldrin et al., 2013), model 2 (Karlsson, 2011)], as well as assessing their advantages and disadvantages, we made the following plan for our research:

1. create a hybrid of both models: take into account all set of confounders, check the model without trend, check all sets of AB, check the model with logarithm as a link function and without link function;
2. introduce the AB consumption as the proportion of patients who received AB;
3. introduce the second step of validation (using test sets).

These goals highlight the importance of considering and incorporating positive aspects of both models in the class to build the most robust model. Class 1 models are defined by the use of DDD as the variable indicating AB use and their great advantage is the inclusion of the AB consumption parameter through the volume or dose (DDD). In the process of using actual consumption data we have to consider the uniformity of consumption of drugs among patients, in order to avoid incorrect parameter estimation.

Second, to apply model 3 of class 2 where AB usage is introduced “by fraction,” it is required to make a number of remarks (D'Agata et al., 2005). The most interesting for us is to consider the dynamics of the total amount of patients colonized by resistant type of strain, or changing the proportion of patients (as a measure of resistance). AB consumption is not included in the model as a key input parameter. The model does not give an analytic representation of the dependence between the number of drug-resistant strains and fractions of patients receiving treatment. However, during the simulation modeling by changing the proportion of patients receiving treatment, we can track the dynamics in the resistant strains and construct a graph of this dependence. The results obtained in this research will allow us not only to test them on the basis of our data, but also to compare the class 1 and class 2 models with each other. If it appears that the segments of patients significantly influence the change resistance, it is reasonable to build a new model based on the class 1. However, the effect of AB is introduced not through the dose, but is accounted for in the segments of patients receiving treatment.

Model 4 has as a key component the number of drug-resistant bacteria. In constructing our model we would want to consider not the number of bacteria but the number of patients diagnosed with the resistant strain, and to slightly change the parameters of the model accordingly. This type of model will be able to be validated using our data and if the conclusions are quantitatively confirmed by our data, it will be useful to incorporate key aspects of the model when developing our own. However, it will be necessary to significantly modify the model, for example, to exclude the part relating to the HCWs as well as the self-contained aspect (patients do not enter or leave the hospital).

Model 5 can be used to inform our model as it gives information on the relative methods and benefits of considering a within-host model or a between-host model, or what one might describe as extrapolating from bacteria to patients. In that perspective, the hospital is seen as the host and the patients as bacteria. According to this new logic, we need to give new definitions to the parameters. Suppose the growth rate of bacteria λ is the change in patient dynamics; the division rate δ is the rate of new patient admissions; the mortality rate μ refers to the number of discharged (recovered) patients. This is also not contrary to the main assumptions of the model, as recovery may be related to the average dose of AB, though we suspect that it would be better to describe this parameter separately, as it was in the first article of this model class examined. One of the distinctions between class 3 and class 1 (with DDD) is that in class 3 there is no influence of previous periods in the development of resistance, i.e., it will be difficult to make a correct prediction for the future situation. Class 3 is more complex because the models in this class are based on biological processes that are difficult to verify in practice.

**We have identified two major decision points for building a robust and pertinent model these two decisions are:**

1. The dichotomy of mathematical and biological approaches.
2. The dichotomy of smoothing or not smoothing data.

The best example of biological approaches comes in class 3 models, which consider such biological mechanisms as mutation, immunity, and horizontal gene transfer. Mathematical models described in class 1 consider aspects of time series; moreover class 1 models consider the influence of AB through the dose (or consumption) parameter, with the length of treatment assessed within this parameter. Class 3 introduces the influence of AB separately through the killing rate, as a coefficient of the mortality of bacteria caused by the action of AB, and the length of treatment. Assessment of the killing rate of AB seems to be difficult in the framework of our study, so we are going to estimate this parameter in another way (using AB dosage and time of recovery). However, in both scenarios smoothing the dosage and consumption parameters needs to be considered. As we understand things, consumption is determined by two parameters: the dosage (mainly, DDD) and the length of treatment, which may distribute unequally amongst patients receiving a drug. It is necessary to understand whether these parameters are important or they just make a model noisy. Perhaps, if AB consumption is introduced into model either through the percentage of patients receiving a drug (as in class 2) or through the number of prescriptions (model 7), the predictive ability of the model will be more accurate. It will be possible to answer this question by creating models with different approaches based on the same data. Moreover, models of class 1 can also be considered from the percentage (of prescriptions), not from the AB consumption. If we verify two different approaches in one model, we will also be able to understand whether smoothing these variables is a beneficial step.

## Conclusions and plans

To conclude, we want to emphasize that the main advantage of any new study over those discussed above is a consequence of the breadth of information and knowledge garnered by reviewing each of the models since they all have positive aspects to them. As a result, we will be able to verify these models using the same set of data. This should allow us to build an optimal model, and to extend the amount of independent models for the following prognosis. Let us recall that suppose that the average (or the weighted average) value of independent prognoses of the process is always more preferable than only one prognosis.

Further work is planned as follows: modify and test these models in accordance with our data and objectives, consider the construction of a model using approaches (regressions, differential equations, etc.) from one class and method of introducing AB into a model from another class check all the variety of models obtained on our data and select the model (or several models) that best describes resistance dynamics (giving the best quality of the forecast). This procedure will help us to select the optimal approach or combination of approaches. Based on the results obtained, we will build a robust and appropriate model for resistance development.

### Conflict of interest statement

This review was formed during the study funded by pharmaceutical Company AstraZeneca. The authors declare that the research was conducted in the absence of any commercial or financial relationships that could be construed as a potential conflict of interest.
